# A Comparison of Carotid Ultrasound and Coronary Optical Coherence Tomography in Assessing Plaque Morphology in Patients with Chronic Coronary Syndrome

**DOI:** 10.1177/00033197251355336

**Published:** 2025-07-30

**Authors:** Mehmet Ali Cetiner, Marc Vorpahl, Melchior Seyfarth, Ulrich Wiederhold, Juliane Sophie Yu, Rojvan Bilgin, Ramazan Gökmen Turan

**Affiliations:** 1 Department of Cardiology, University of Witten/Herdecke, Germany; 2 Foundation of the Cellitinnen to the Holy Maria, Bergheim, Nordrhein-Westfalen, Germany

**Keywords:** imaging, optical coherence tomography, carotid ultrasound, atherosclerosis

## Abstract

Atherosclerosis is a predominant cause of cardiovascular events and the accurate assessment of plaque morphology is crucial for risk stratification and treatment planning. Carotid ultrasound (CUS) and coronary artery optical coherence tomography (OCT) are valuable imaging modalities for evaluating atherosclerotic plaques. This study aimed to compare the reliability of CUS and OCT in assessing plaque morphology and atherosclerosis in patients with chronic coronary syndrome. Forty-six patients with chronic coronary syndrome underwent OCT to evaluate coronary plaques and CUS to assess carotid plaques. The findings showed that both imaging techniques detected the presence of plaques, with OCT providing more detailed information on coronary plaque morphology compared with CUS. OCT and CUS measurements demonstrated significant correlations in lumen stenosis, plaque sizes, and representations of calcified plaques. Furthermore, distinct differences were observed in plaque distribution and characteristics between the coronary and carotid arteries. Certain cardiovascular risk factors such as diabetes, arterial hypertension, and obesity were associated with specific plaque properties identified by OCT. This study underscores the complementary roles of OCT and CUS in assessing atherosclerosis and plaque vulnerability in patients with chronic coronary syndrome, providing valuable insights for clinical management and treatment strategies.

## Introduction

Coronary artery disease (CAD) is not only the most common cardiovascular disease but also the leading cause of death worldwide. In the past, non-invasive procedures such as cardiac stress tests were used for CAD diagnosis. However, invasive imaging modalities have become increasingly important in recent years. Techniques like intravascular ultrasound (IVUS), optical coherence tomography (OCT), and near-infrared spectroscopy (NIRS) allow for a more detailed assessment of plaque presence and morphological characteristics.^1^

OCT, an invasive intravascular imaging technique, utilizes infrared light to capture high-resolution images with an image resolution of approximately 10 to 15 μm. Over time, OCT has evolved into a catheter-based imaging technology providing insights into vascular biology and guidance for percutaneous intervention (PCI). OCT enables precise measurement of plaque fibrous cap thickness, correlating strongly with histology, and offers detailed insights into plaque composition, including inflammatory cells, lipid pools, and calcifications, as well as intraluminal thrombi. OCT allows for clear distinction of all vascular layers. Disadvantages of OCT include high costs, limited availability, invasiveness, and the requirement for contrast medium administration. However, advancements in technology have led to achieving high image resolution with minimal contrast medium usage.^2^

Traditional diagnostic methods such as carotid vessel ultrasonography (CUS) continue to be important, especially in outpatient settings, as the carotid arteries are significant sites of atherosclerosis manifestation. CUS enables visualization of atherosclerotic plaques in extracranial brain vessels and provides information on plaque morphology, including calcification, lipid content, intraplaque hemorrhage, plaque area, plaque volume, and intima-media thickness (IMT), serving as crucial determinants of atherosclerosis.^3^

Over the past three decades, carotid sclerosis and CAD have shown increasing prevalence. Recent data suggest that the development of atherosclerotic plaques, the fundamental component of both diseases, shares similar features and mechanisms regardless of the affected vessel. Nonetheless, disparities exist in plaque morphology and properties between coronary and carotid artery disease. Factors like plaque erosions, calcifications, fibrous cap thickness, and macrophage accumulation may vary. Additionally, the surrounding tissue of both arteries may differently influence plaque biology.^4^

The objective of this study is to assess the correlation between coronary artery atherosclerosis detected by OCT and concurrent carotid atherosclerosis based on sonographic criteria. Furthermore, the study aims to investigate and compare the relationships between epidemiological factors and plaque properties identified by both imaging methods. In the case of a weak or negative correlation between the two methods, the study aims to determine to what extent carotid ultrasound can identify structural changes in the vascular tree compared with coronary artery OCT.

## Materials and Methods

### Study Population

The study was designed as a prospective, observational, cross-sectional, single-center study conducted over a 15-month period. Forty-six patients were enrolled between January 1, 2021, and March 31, 2022, who were receiving treatment at the cardiology department, for chronic coronary syndrome with a guideline-compliant indication for cardiac catheterization and OCT measurement. In addition to OCT, CUS was carried out with blinded analysis for study purposes.

### Imaging Protocols

Cardiac catheterizations were performed using the Allura FD 15 X-ray system (Philips Medical Systems^®^, Eindhoven, The Netherlands). OCT examinations were conducted using the Optis Integrated OCT system (Abbott Cardiovascular^®^, Plymouth, USA). The recorded sections during the examination were analyzed offline using an application (Angle Meter^®^ App, Smart Tool Factory Inc., Oklahoma City, Oklahoma, USA).

Obesity was defined according to WHO (World Health Organization) – Classification: follows; Overweight (not obese), if BMI is 25.0 to 29.9. Class 1 (low-risk) obesity, if BMI is 30.0 to 34.9. Class 2 (moderate-risk) obesity, if BMI is 35.0 to 39.9. Class 3 (high-risk) obesity, if BMI is ≥40.^5^ Any known condition of narrowed arteries that reduces blood flow to the limbs was accepted as peripheral artery disease. The coronary arteries are coded as left anterior descending artery (LAD), the circumflex artery (RCX) and right coronary artery (RCA).

Plaque types identified by OCT were categorized as fibrous if appearing as high-signal, homogeneous regions with low signal attenuation. Calcified plaques presented as low-signal, homogeneous areas with sharp borders and low signal attenuation, while atheromatous plaques manifested as low-signal regions with diffuse edges and high signal attenuation within a lesion. Any plaque with a fibrotic component was defined as fibrosis positive in our study. This was also the case for calcific and atheromatous plaques.

Microvessels were defined as low-signal cavities with a diameter of 50 to 150 µm and a trajectory parallel to the lumen, visible on three consecutive OCT cross-sectional images. The differences between the original vessel area and diameter and the current vessel lumen and diameter were recorded as area stenosis and diameter stenosis.

Calcium and lipid arc refer to the angular extent of calcium and lipid deposits within a coronary artery, measured on an OCT image. The quadrants of calcium and lipid were calculated by dividing the 360º vessel circumference into 90º. Each 90º of the measured angles corresponded to a quadrant.

Calcified plaques with <3 quadrants of the circumference (<270°) or a calcium depth of <0.90 mm were classified as eccentric, while those with ≥270° of circumference or a depth of ≥0.90 mm were classified as concentric. Calcium localization was considered superficial if present at the tunica intima-lumen interface, and deep if located within the medial-adventitial border or closer to the tunica adventitia than the lumen.^6^

Carotid duplex sonography was performed in a supine position using an Epiq 7c (Philips Medical Systems^®^, Eindhoven, The Netherlands) ultrasound device. Similar to OCT measurements, vessel and plaque measurements were conducted in a horizontal section using the length and contour functions. Carotid plaques were defined according to the Mannheim consensus^7^ and described based on their echogenicity as fibrotic (isoechoic), atheromatous (echolucent), and calcified (hyperechogenic with acoustic shadows).

Carotid plaques were classified as focal if the IMT was between 1.1 and 1.5 mm, and diffuse if it was ≥1.5 mm.^8^ Carotid plaques were categorized into five types according to the pattern of echolucency and echogenicity using the Gray–Weale classification system.^9^ The Gray–Weale classification system is used to categorize carotid artery plaques based on their ultrasound characteristics, specifically their echogenicity (how bright or dark they appear on the ultrasound). It is a subjective system, relying on visual inspection of ultrasound images to categorize plaques.

### Data Analysis

A physical examination was conducted following detailed verbal and written information. Patient data regarding cardiovascular risk profiles were collected fasting and laboratory values were determined within 24 hours before the catheter examination. Detailed information on cardiovascular history and medications was recorded. Patients who had not used nicotine or nicotine products in the past 12 months were categorized as ex-smokers. Any coronary artery with an angiographic stenosis exceeding 50% was defined as diseased.

### Statistical Analysis

The anonymized raw data were analyzed using the IBM SPSS Statistics program version 22.0 (IBM Inc., Armonk, NY, USA). Descriptive analysis involved calculating absolute and relative frequencies, means, standard deviations, medians, and quartiles based on the data type. Categorical variables were presented as numbers (percentage). Pearson correlation analysis was used to describe and measure the relationship between two numerical variables with homogeneous and normal distribution. Corresponding hypothesis tests for univariable group differences were carried out by chi-square tests, Fisher’s exact tests (or Fisher–Freeman–Halton tests). In independent samples, if the prerequisites for a parametric method were not met, either a Mann–Whitney *U* test or a Kruskal–Wallis test was used. For combined samples, a paired *t*-test was used.

The aim of this study was to determine the correlation of coronary OCT and carotid duplex sonography in atherosclerosis and to obtain a significant result (*P* < .05) with sufficient power (80%) to capture at least a correlation coefficient of .4. Therefore, the minimum sample size required for this study was 46. The calculation formula was based on a two-sided examination.

## Results

### Patient Characteristics

Among the 46 patients enrolled in the study, 34 (73.9%) were male, and 12 (26.1%) were female. The main cardiovascular risk factors of the participants are presented in Table 1. The majority of patients presented with severe CAD, with about 85% having a history of coronary interventions. As 67% of patients had two or three vessel disease, 17% of the patients had a left main stem disease.

**Table 1.** Cardiovascular Disease Risk Factors.

		n	%
Diabetes mellitus		14	30.4
Hyperlipidemia		42	91.3
Obesity	Overweight	26	56.5
	Class 1	9	19.6
	Class 2	2	4.3
	Class 3	0	0.0
Peripheral artery disease		6	13.0
TIA and/or stroke		3	6.5
Arterial hypertension		33	71.7
Family history of coronary artery disease		27	58.7
Nicotine abuse	Active	26	56.5
	No/ex-nicotine abuse	20	43.5
Hyperuricemia		4	8.7

While a history of diabetes mellitus was classified as diabetes mellitus, obesity was defined according to BMI values and the WHO classification. Patients who had not consumed nicotine or nicotine products (including e-cigarettes) in the past 12 months were classified as ex-smokers. Any type and amount of nicotine intake in the past 12 months was classified as an activator of nicotine abuse.

Abbreviation: TIA, transient ischemic attack.

### Plaque Presence

A total of 86 OCT images were obtained from 46 patients, using a contrast medium volume of 32 ± 10 mL; 167 carotid plaques were observed using CUS in 44 out of 46 patients, with prevalent plaques in the bulb area, followed by the ACI (internal carotid artery) and ACC (common carotid artery). Notably, two patients had no carotid artery plaques despite having significant CAD.

### Comparison of Imaging Modalities

In OCT examinations, the presence of high calcium content in coronary plaques up to 90% was notable. Furthermore, approximately half of the plaques exhibited microvessels, suggesting ongoing revascularization within the plaque (Table 2).

**Table 2.** Morphological Features of Coronary Plaques Using OCT.

		n	%
OCT-vessel affected	LAD	18	39.1
	RCX	7	15.2
	RCA	21	45.7
OCT plaque-property fibrosis	Fibrosis positive	39	84.8
	Negative	7	15.2
OCT plaque-property atheromatous	Atheroma positive	39	84.8
	Negative	7	15.2
OCT plaque-property calcified	Calcified plaque positive	42	91.3
	Negative	4	8.7
Microvessels	Microvessels positive	21	45.7
	Negative	25	54.3

Abbreviation: OCT, optical coherence tomography.

Compared with OCT, CUS provided limited information on atherosclerotic plaque features. (Table 3).

**Table 3.** Morphological Features of Carotid Plaques in CUS.

		n	%
Largest plaque location	Neither side	2	4.3
	Right side	28	60.9
	Left side	16	34.8
Plaque-type	Focal	17	37.0
	Diffuse	27	58.7
Fibrotic plaque	Neither side	3	6.5
	Unilateral fibrosis	10	21.7
	Bilateral fibrosis	33	71.7
Atheromatous plaque	Neither side	14	30.4
	Unilateral Atheroma	14	30.4
	Bilateral Atheroma	18	39.1
Calcified plaque	Neither side	8	17.4
	Unilateral	12	26.1
	Bilateral	26	56.5
Gray–Weale classification	No plaque	2	4.3
	Type 1	16	34.8
	Type 2	13	28.3
	Type 3	12	26.1
	Type 4	3	6.5

Abbreviation: CUS, carotid ultrasound.

### Correlations Between the Imaging Findings

On average, the measured coronary plaques caused an area decrease of 48.9% ± 13.4% and a diameter decrease of 27.8% ± 11.0% in OCT, while in CUS, these values were 40.5% ± 15.3% and 33.0% ± 11.1%, respectively. Furthermore, the average maximum calcium angle was significantly lower in ultrasound compared with OCT (81.9% vs 120.8%). Calcium was primarily localized superficially (57.9%) in CUS and deeply (78.3%) in OCT.

Statistical analysis revealed that age was the only significant predictor among the demographic parameters for OCT and CUS. With advancing age, a notable increase was observed, particularly in calcium measurements in OCT and diameter stenosis in CUS.

The association analysis indicated a strong relationship between diabetes mellitus and fibrosis, arterial hypertension with microvessel formation, and obesity with calcium localization in OCT, while no significant correlations were found between CUS plaque characteristics and cardiovascular risk factors (Table 4).

**Table 4.** Association of Cardiovascular Risk Factors with OCT.

	OCT plaque							
	Fibrosis	Atheroma	Calcified plaque	Microvessel	Lipid arc	Calcium arc	Calcium type	Calcium localization
*P-value*								
DM	.01*	.60	.49	.37	.20	.53	.41	.44
Arterial hypertension	.35	.45	.44	.04*	.06	.39	.46	.38
Hyperlipidemia	.50	.56	.62	.37	.54	.43	.62	.62
Obesity	.31	.49	.29	.40	.19	.34	.55	.03*
Family history	.93	.64	.95	.43	.06	.11	.48	.36
Nicotine abuse	.43	.22	.08	.26	.40	.07	.29	.42

Abbreviations: OCT, optical coherence tomography; DM, diabetes mellitus.

* The correlation is significant at the 0.05 level (two sided).

Concerning vascular measurements and plaque properties, a significant relationship between OCT and CUS was found in the *t*-test for dependent samples. However, there was no difference between the measurements in the correlation analysis. While the *t*-test focuses on mean differences between groups, the correlation assesses the relationship between variables. Also, the *t*-test can detect small differences in means according to the sample size, even if the variables are not strongly correlated.

## Discussion

One of the key features of the present study was the focus on patients with chronic coronary syndrome and severe CAD.

Cohen et al. conducted a study involving 150 subjects who underwent computed tomography angiography (CTA) and carotid ultrasound on the same day.^10^ The subjects underwent both CTA and carotid ultrasound on the same day. Carotid plaque was present in 71.3% (n = 107), whereas the presence of at least 1 coronary artery with disease on CTA was present in 57.1% (n = 84). The authors found that carotid plaque was present in 47.6% (30 of 63) of subjects with a calcium score of 0 and 88.5% (77 of 87) of subjects with a calcium score >0 (*P* = .0001)

On the other hand La Grutta et al^11^ compared coronary and carotid artery atherosclerosis in patients undergoing noninvasive cardiac CT for suspected stable CAD, noting differences in plaque characteristics between the coronary tree and carotid arteries with respect to lesion severity, position along the vessel course, and composition of plaque. In this study the shape of the plaques was eccentric in 96% of cases (n = 66) in the coronary district and in 92% (n = 46) in carotid arteries (*P* > .05). Coronary plaques presented a calcified pattern in 25% (n = 17), non-calcified in 19% (n = 13), and mixed in 56% (n = 9), while carotid plaques were calcified in 8% (n = 4), non-calcified in 8% (n = 4), and mixed in 84% (n = 42). The severity of disease was similar in the two arterial districts when calcium score is 0 (*P* > .05), however, significantly differed when calcium score was 1 to 100, 101 to 400, and >400 (*P* < .05). Although similar regarding study design, our investigation utilized OCT, which is known for its higher sensitivity in imaging coronary arteries.

A significant finding was the presence of coronary plaques in all patients via OCT, yet two patients showed no carotid plaques despite severe CAD, highlighting differences in plaque morphology between the carotid and coronary arteries. Additionally, the pathology of plaque formation differs between these vessels, possibly influenced by variations in endothelial shear stresses and perivascular fat distribution.^4^

In the present study, the detection rate of both coronary and carotid artery plaques among patients with severe CAD was notably high, underscoring the predictive value of discovering plaques in both regions. The presence of varied morphological features in carotid ultrasound, such as IMT and plaque surface irregularity, has been shown to provide enhanced risk prediction beyond conventional factors.^12^

OCT, known for its ability to detect plaque morphology, has identified features associated with plaque vulnerability, including microvessels, which serve as predictors of lumen narrowing. Vulnerable plaques containing a large lipid core, thin fibrous cap, and neoangiogenesis are prone to rupture, leading to thrombosis and acute coronary events.^13,14^ The presence of microvessels in almost half of the patients in this study underscores the severity of coronary disease and plaque vulnerability.

Studies associating OCT images with histology have shown robust sensitivity and specificity for plaque classification.^15^ Similarly, the present study identified a high prevalence of fibrotic, lipid-rich, and calcified plaques in line with the robust nature of OCT in plaque characterization.

The Multi-Ethnic Study of Atherosclerosis (MESA) study highlighted the predictive value of coronary calcium and carotid plaque, emphasizing their role in predicting cardiovascular events.^16^ In this Study with 6779 participants with a mean age of 62.2 years, the 49.9% of patients revealed coronary artery calcium and 46.7% carotid plaques. After 9.5 years, 538 cardiovascular disease (CVD) events, 388 coronary heart disease (CHD) events, and 196 stroke/transient ischemic attacks were observed. Calcium presence in the coronary artery was a stronger predictor of incident CVD and CHD than carotid ultrasound measures. Compared with traditional risk factors, *c*-statistics for CVD (*c* = 0.756) and CHD (*c* = 0.752) increased the most by the addition of coronary artery calcium presence (CVD, 0.776; CHD, 0.784; *P* < .001) followed by carotid plaque presence (CVD, *c* = 0.760; CHD, *c* = 0.757; *P* < .05). Compared with risk factors (*c* = 0.782), carotid plaque presence (*c* = 0.787; *P* = .045) but not coronary artery calcium (*c* = 0.785; *P* = .438) improved prediction of stroke/transient ischemic attacks.

Calcification in coronary arteries can arise due to atherosclerosis or medial arteriosclerosis. While atherosclerotic calcification is mainly intimal, medial calcification is linked to advanced age and conditions like diabetes and chronic kidney disease, contributing to arterial stiffness and elevated cardiovascular risks.^17,18^

The AWHS (Femoral and Carotid Subclinical Atherosclerosis Association with Risk Factors and Coronary Calcium) Study found a higher rate of deeply located calcified plaques in the coronary tree with a more superficial placement in carotid arteries, likely influenced by the patients’ heightened cardiovascular risk profiles.^19^ The studied sample included 1423 men with a mean age of 51.0 ± 3.7 years. At least 1 atherosclerotic lesion was present in 1020 participants (72%). Plaques were detected by ultrasound in 65% of participants (34% in the carotid and 54% in the femoral arteries), and 38% had positive coronary calcification. Current smoking was the independent factor most strongly associated with femoral and carotid plaques, whereas hypertension was the risk factor most strongly associated with the presence of coronary calcium. Only 76 participants had diabetes, and the adjusted association did not show statistical significance for diabetes in the carotid or coronary territories, whereas it was significant for femoral plaques. The accumulation of risk factors had a strong effect on femoral atherosclerosis, which was the territory that drove most of the rise in the association of risk factors with atherosclerotic presence in any territory. As a conclusion subclinical atherosclerosis was highly prevalent in this middle-aged male cohort. Association with risk factors and positive coronary artery calcium score was stronger in femoral than carotid arteries.

The occurrence of calcium deposits in coronary arteries is age and sex-dependent, with higher prevalence in older males. Our findings align with previous studies, showing a significant increase in calcium measurements with advancing age using OCT.^20^ Furthermore, findings in diabetic patients revealed pronounced fibrosis in coronary arteries, particularly notable on OCT images. In contrast, arterial hypertension was associated with neovascularization and microvessel formation detected by OCT. Notably, obese individuals exhibited deeper calcium plaque presence, underscoring obesity’s impact on plaque localization. While both coronary OCT and CUS detected plaque presence in severe CAD patients, CUS was found to have limited capabilities in assessing plaque morphology and severity compared with OCT. Despite the correlations observed between OCT and CUS in detecting lumen stenosis, plaque sizes, and calcified plaques, the two methods showed discrepancies in identifying fine morphological features. The major limitation of the study is the small sample size.

Intracoronary imaging methods, including OCT, are a rapidly developing field and time is needed for the findings to be embedded in the scientific literature.

## Conclusion

Key results of this study include:

– Both coronary OCT and CUS revealed high plaque presence rates in patients with severe CAD.
– Dissimilarities were observed in plaque distributions and compositions between the carotid and coronary arteries.
– Coronary OCT showed superior efficacy in identifying plaque characteristics in arteries compared with CUS.
– Calcium localization varied between the two imaging methods, possibly influenced by patient cardiovascular risk profiles.
– Associations were found between demographic factors and certain plaque features.
– Despite correlations, OCT and CUS displayed discrepancies in fine morphological assessments.

In conclusion, while CUS offers some insight into plaque presence in severe CAD patients, OCT remains the superior modality for in-depth plaque characterization.
